# Surfactant Tween 20 Controlled Perovskite Film Fabricated by Thermal Blade Coating for Efficient Perovskite Solar Cells

**DOI:** 10.3390/nano12152651

**Published:** 2022-08-02

**Authors:** Kun-Mu Lee, Shun-Hsiang Chan, Chang-Chieh Ting, Shih-Hsuan Chen, Wei-Hao Chiu, Vembu Suryanarayanan, Jen-Fu Hsu, Ching-Yuan Liu, Ming-Chung Wu

**Affiliations:** 1 Department of Chemical and Materials Engineering, Chang Gung University, Taoyuan 33302, Taiwan; cgu.shihhsuanchen@gmail.com; 2 Green Technology Research Center, Chang Gung University, Taoyuan 33302, Taiwan; 3 Division of Neonatology, Department of Pediatrics, Chang Gung Memorial Hospital, Linkou, Taoyuan 33305, Taiwan; hsujanfu@cgmh.org.tw; 4 Center for Reliability Sciences and Technologies, Chang Gung University, Taoyuan 33302, Taiwan; d000017236@cgu.edu.tw; 5 Department of Chemical and Materials Engineering, National Central University, Jhongli District, Taoyuan 32001, Taiwan; kero5206@gmail.com; 6 Electroorganic and Materials Electrochemistry Division, CSIR-Central Electrochemical Research Institute, Karaikudi 630003, India; vidhyasur@yahoo.co.in

**Keywords:** perovskite solar cells, surfactant Tween 20, thermal-assisted blade coating, power conversion efficiency

## Abstract

In recent years, additive engineering has received considerable attention for the fabrication of high-performance perovskite solar cells (PSCs). In this study, a non-ionic surfactant, polyoxyethylene (20) sorbitan monolaurate (Tween 20), was added as an additive into the MAPbI_3_ perovskite layer, and the thermal-assisted blade-coating method was used to fabricate a high-quality perovskite film. The Tween 20 effectively passivated defects and traps in the MAPbI_3_ perovskite films. Such a film fabricated with an appropriate amount of Tween 20 on the substrate showed a higher photoluminescence (PL) intensity and longer carrier lifetime. At the optimal concentration of 1.0 mM Tween 20, the performance of the PSC was apparently enhanced, and the champion PSC demonstrated a PCE of 18.80%. Finally, this study further explored and compared the effect on the device performance and ambient stability of the MAPbI_3_ perovskite film prepared by the spin-coating method and the thermal-assisted blade coating.

## 1. Introduction

Over the past decade, metal halide perovskite solar cells (PSCs) have resulted in tremendous interest in the next generation of solar cell technologies, benefiting from their high absorption coefficient [1,2,3,4,5], ever-increasing photoelectric characteristics [6,7], low-cost materials [8,9], simple fabrication process [10], and so on. The typical three-dimensional (3D) perovskite structure is represented by the ABX_3_ structure, where A is a monovalent cation, B is a divalent metal cation, and X is a halide anion [11]. Because the perovskite films are known to exhibit unavoidable defects that act as charge recombination centers, the defects could deteriorate charge carrier transport and collection and hinder the performance improvement of the devices. Recently, many scientists have devoted themselves to studying PSCs by optimizing the perovskite layer and the interface between each layer to achieve high power conversion efficiency (PCE), which has exceeded 25% in the last decade.

To obtain high-performance and stable perovskite devices, controlling the morphology [12,13], grain boundary [14], grain size, charge recombination [15], and the density of the defect states in the perovskite film are necessary [16]. Therefore, how to prepare a high-quality, uniform, and pinhole-free perovskite film is an important issue. Currently, there are many techniques such as composition adjustment [17], process parameter control, and the use of additive [18] for preparing high-quality perovskite film. Additive engineering in perovskite films has been well investigated in PSCs. Among them, the polymeric molecules are usually used as an additive, because the specific functional groups and lone pair electrons on oxygen, sulfur, or nitrogen as Lewis bases in the main or side chain can effectively passivate defects of the perovskite [19,20,21,22]. Moreover, the polymers with a long chain can not only act as scaffolds of perovskite crystals but also cross-linking the perovskite grains to obtain the large crystal grains and smooth surface of the perovskite films [23,24]. Some of the literature has shown that adding a small amount of polymer to perovskites can effectively passivate defects and traps. [25,26].

The lab-scale PSCs, which show great achievement in device efficiency, typically use spin-coating techniques in an inert glovebox environment. However, the spin-coating technique has a low throughput, wastes a large amount of precursor solution, and is unsuitable for preparing large-area PSCs. To enable continuous manufacturing, many large-area coating methods have been developed, including slot die [27], blade coating [28,29,30], inkjet printing [31,32], vacuum deposition [33], and spray coating [34]. Among these scalable deposition technologies, the blade-coating method received more attention because it is compatible with roll-to-roll fabrication. In addition, the blade-coating method has the advantage of being a simple, cost-efficient, and low-temperature process. Unlike the spin-coating method, the blade-coating method can control the substrate temperature, the gap between the blade and substrate, and the coating speed for large-grain-size perovskite film [35].

In this study, a non-ionic surfactant, polyoxyethylene (20) sorbitan monolaurate (Tween 20), was added to the perovskite precursor solution [36]. The MAPbI_3_ perovskite films were fabricated by the thermal-assisted blade-coating method. The results show that the crystalline domain size of the MAPbI_3_ perovskite film can be controlled by adding Tween 20. Adding an appropriate amount of Tween 20 into the MAPbI_3_ perovskite film also significantly enhances the radiative recombination. At the optimal Tween 20 (1.0 mM) concentration, the champion PSC demonstrated a PCE of 18.80%. This work gives an insight into the effect of surfactants in MAPbI_3_ perovskite devices produced by the thermal-assisted blade-coating method for the large-area fabrication process.

## 2. Materials and Methods

### 2.1. Preparation of Materials

All reagents were analytical grade and used as received without further purifications. For preparation of the dense TiO_2_ precursor solution, 1.0 mL of titanium diisopropoxide bis(acetylacetonate) was added in 39.0 mL of ethanol (CH_3_CH_2_OH, >99.8%, Sigma-Aldrich, St. Louis, MO, USA). The synthesis of TiO_2_ paste was based on our previous study [37]. Briefly, 25.0 g of titanium isopropoxide (Ti(OCH(CH_3_)_2_)_4_, >97%, Sigma-Aldrich, St. Louis, MO, USA) was added in 10.0 mL of 2-propanol ((CH_3_)_2_CHOH, IPA, >99.8%, STAREK, Taipei, Taiwan) while stirring. Next, TTIP/IPA solution was dropped into 90.0 mL of 3.5 M acetic acid (CH_3_COOH, >99.7%, Sigma-Aldrich, St. Louis, MO, USA), cooled in an ice bath for 12 h, and then heated at 80 °C for 8 h. The pale yellow solution was transferred into an autoclave at 170 °C for 6 h to obtain TiO_2_ nanoparticles. A total of 23.0 wt% of TiO_2_ nanoparticles was diluted with α-terpineol (C_10_H_18_O, 90%, Merck, Darmstadt, Germany) and ethyl cellulose (ethoxyl content 48%, 22 cps, Acros Organics, Geel, Belgium). The pristine perovskite solution of 1.5 M CH_3_NH_3_I (MAI, >98%, FrontMaterials, Taipei, Taiwan) and 1.5 M lead iodide (PbI_2_, 99.9985%, Alfa Aesar, Tewksbury, MA, USA) in a mixed solvent of DMSO (99.9%, ECHO)/GBL (≥99%, Acros Organics, Geel, Belgium) (90/10 *v*/*v*) was prepared. For Tween 20 perovskite solution, various concentrations of Tween 20 were added to the MAPbI_3_ perovskite precursor solution, including 1.0, 2.0, and 3.0 mM, respectively. The preparation of hole transport material (HTM, spiro-OMeTAD, FrontMaterials, Taipei, Taiwan) was based on the previous work [38].

### 2.2. Fabrication of the MAPbI_3_ Perovskite Solar Cells

The FTO glass (10 × 10 cm, 7 Ω/square, Ruilong, Miaoli, Taiwan) was ultrasonically cleaned with deionized water, acetone, IPA, followed by 10 min of UV–ozone treatment. Then, the dense TiO_2_ was deposited on the FTO glass at 450 °C using the spray pyrolysis method. To deposit the mesoporous TiO_2_ (mp-TiO_2_) layer, the TiO_2_ paste was on top of the dense TiO_2_ using the screen-printing method, followed by 30 min of calcination at 500 °C to form the mp-TiO_2_ layer. For the fabrication of the MAPbI_3_ perovskite layer, a 2.5 × 5.0 cm FTO/dense TiO_2_/mp-TiO_2_ was preheated to 130 °C. A total of 30.0 μL of perovskite precursor solution was dropped on the top of the mp-TiO_2_ layer under ambient atmosphere (30–40 %RH). Then, at 130 °C, the metal blade (ZUA 2000, Zehntner, Sissach, Switzerland) scraped excess solution for 3 min at a coating speed of 3.0 cm/s and a blade gap of 300 μm. The HTM solution was spin-coated on the perovskite layer at 2000 rpm for 30 s. A 100 nm thick silver electrode was deposited on the HTM using thermal evaporation with a 0.09 cm^2^ metal mask.

### 2.3. Characterizations

The surface morphology of MAPbI_3_ perovskite films was measured by atomic force microscopy (AFM) (Multimode2-U-NSV, Bruker, Billerica, MA, USA). Field-emission scanning electron microscopy (FE-SEM) images were obtained using a HITACHI (Tokyo, Japan) su8010 to observe samples’ surface and cross-section morphology. XRD analysis was obtained using an X-ray diffractometer (D2 phaser, Bruker, Billerica, MA, USA) with Cu Kα (λ = 1.5418 Å) radiation. An ultraviolet–visible spectrophotometer (V-730, Jasco, Tokyo, Japan) was used to measure the optical properties of the MAPbI_3_ perovskite film. The photoluminescence (PL) spectra and time-resolved photoluminescence (TRPL) spectra were recorded with a continuous 532 nm diode laser (LDH-D-TA-530, PicoQuant, Berlin, Germany). The TRPL–decay curves were recorded by a time-correlated single-photon counting (TCSPC) (TimeHarp 260, PicoQuant, Berlin, Germany) spectrometer. The current density–voltage (J-V) performances of various PSCs were measured using a digital source meter (Keithley 2400 Series, Keithley Instruments Inc., Cleveland, OH, USA) under AM 1.5 G simulated solar illumination (100 mW/cm^2^, YCSS-50, Yamashita Denso Corp., Chiba, Japan). The light intensity was calibrated with a silicon reference cell (BS-520B, Bunkoukeiki Co., Ltd., Tokyo, Japan) with a KG-5 filter. To ensure the reliability and repeatability of data, average photovoltaic parameters of each PSC device were obtained from 18 devices. The external quantum efficiency (EQE) spectra were recorded from 300 to 900 nm using an IPCE spectrometer (EQE-R-3011, Enli Technology Co. Ltd., Kaohsiung, Taiwan).

## 3. Results and Discussion

**Figure 1:**
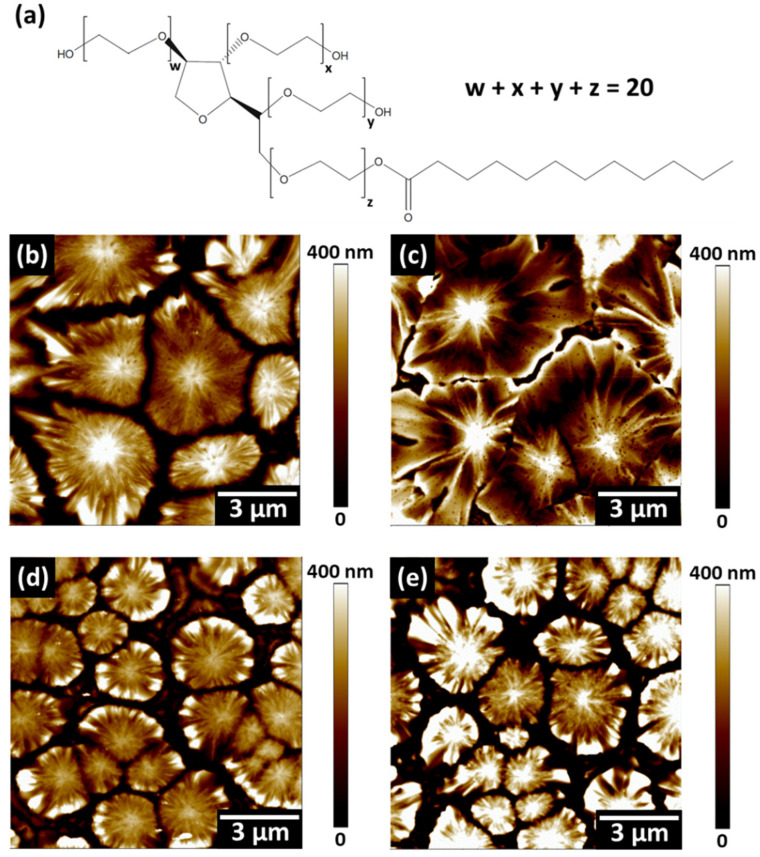
(**a**) Chemical structure of the surfactant Tween 20 and the AFM images of perovskite films with various Tween 20 concentrations, including (**b**) 0.0, (**c**) 1.0, (**d**) 2.0, and (**e**) 3.0 mM.

The surface morphologies of various MAPbI_3_ perovskite films coated on FTO-coated glass/TiO_2_ were characterized by AFM. The chemical structure of the surfactant Tween 20 is shown in Figure 1a. The large-sized perovskite spherulite films can be prepared using the blade-coating method due to the rapid solvent evaporation during the heating [30,39]. The spherulites can be observed in all the perovskite films, as shown in Figure 1b–e, and their RMS and Rs values are also summarized in Table S1. The spherulite size of the pristine perovskite film (0.0 mM) is ~4.0 μm (Figure 1b). When the concentration of the Tween 20 reached 1.0 mM, the size of the spherulites reached ~6.0 μm (Figure 1c), which indicated that the small amount of the Tween 20 could increase the crystal size and decrease the grain boundaries. When the Tween 20 concentration was further increased to 2.0 and 3.0 mM, the spherulite size reduced significantly (Figure 1d,e). The long-chain alkyl groups present in Tween 20 can bridge the perovskite grains by forming a polymer-perovskite composite cross-linker, which produces a large crystal size, defect passivation, and the grain boundaries of the perovskite films. However, the excess Tween 20 addition causes restricted spherulite growth [21,22,40]. In addition, the most uniform surface is obtained by adding 1 mM Tween 20 into the perovskite precursor from the Ra and RMS result of the AFM measurement.

**Figure 2:**
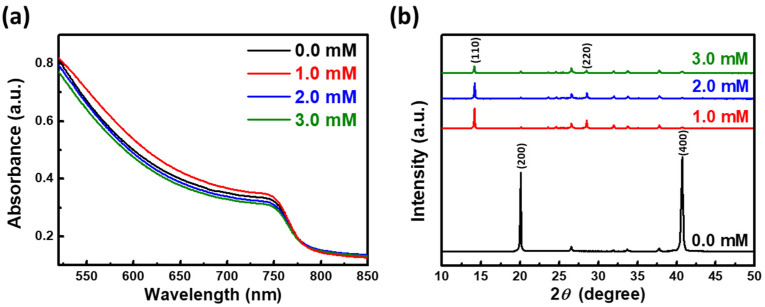
(**a**) UV–vis absorption spectra and (**b**) XRD patterns of pristine, 1.0, 2.0, and 3.0 mM Tween 20 perovskite films.

Figure 2a shows the UV–vis absorption spectra of pristine, 1.0, 2.0, and 3.0 mM Tween 20 perovskite films. The absorbance of the 1.0 mM Tween 20 perovskite film was enhanced due to the large-sized perovskite spherulites. However, the absorbance of the 2.0 and 3.0 mM Tween 20 perovskite films were reduced, which could be attributed to the reduction size of the spherulite sizes [41]. The XRD patterns of the four MAPbI_3_ perovskite films are shown in Figure 2b (the standard XRD patterns of MAPbI_3_ from the database are also shown in Figure S1). The pristine perovskite film showed two diffraction peaks which could be assigned to the (200) and (400) planes. The same results also appeared in our previous work [30]. Here, the fabrication process of the perovskite film affected the preferred crystal orientation of the perovskite, especially during a high-temperature process. It is worth noting that when the Tween 20 concentration was 1.0 mM, the peaks of the (200) and (400) planes were substantially reduced and replaced by the peaks of the (110) and (220) planes. These results showed that adding non-ionic surfactants with long alkyl chains and hydroxyl groups to perovskite films could tune their crystallinity behavior. The hydroxyl group has hydrophilic properties that support the crystal growth of perovskite [42]. On the other hand, the long alkyl chains with hydrophobic properties wrap around the perovskite crystals [43]. However, when excessive Tween 20 was added to the perovskite film, the crystallinity and grain size decreased (Figure S2). The growth of the spherulites was limited because the Tween 20 surrounded the spherulites.

**Figure 3:**
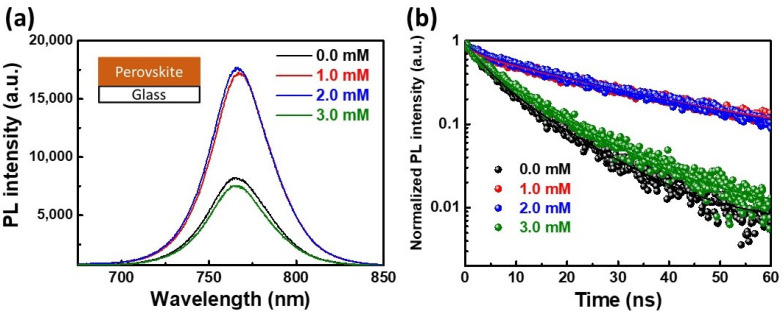
(**a**) PL and (**b**) TRPL spectra of pristine, 1.0, 2.0, and 3.0 mM Tween 20 perovskite films.

The steady-state photoluminescence (PL) spectra of various MAPbI_3_ perovskite film/glass samples were also investigated in detail. As indicated in Figure 3a, the 1.0 and 2.0 mM Tween 20 perovskite films showed a higher PL peak intensity under the same excitation condition due to the reduction in non-radiative losses in the perovskite film. The corresponding time-resolved photoluminescence (TRPL) decay plots were performed to estimate the charge carrier dynamics of various MAPbI_3_ perovskite films (Figure 3b). Herein, a 765 nm single-wavelength light was used as an excitation source. The PL–decay curves were fitted with a bi-exponential decay model [44]:

(1) $$F\left(t\right)=A\exp\left(-\frac{t}{\tau_{1}}\right)+B\exp\left(-\frac{t}{\tau_{2}}\right)$$

where A and B are the weight fractions, $\tau_{1}$ and $\tau_{2}$ are the fast decay lifetime and the slow decay lifetime, respectively. For the average decay lifetime ($\tau_{\mathrm{avg}}$) calculation, the equation is shown below, and the results are listed in Table 1.

(2) $$\tau_{\mathrm{avg}}=\left(A\tau_{1}+B\tau_{2}\right)/\left(A+B\right)$$

The $\tau_{\mathrm{avg}}$ values of the 1.0 and 2.0 mM Tween 20 films increased to 22.5 and 21.5 ns, respectively. According to the above results, the appropriate amount (1.0 mM) of Tween 20 could form a large crystal size and passivate the defect and grain boundaries of the perovskite films, which can suppress carrier trapping, recombination, and significantly improve the slow decay process [40].

**Figure 4:**
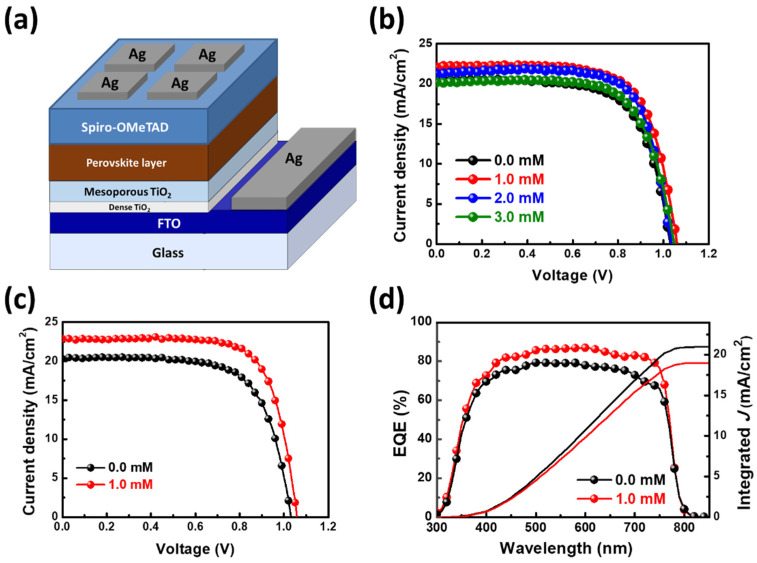
(**a**) The schematic diagram of PSC. (**b**) J-V curves of pristine, 1.0, 2.0, and 3.0 mM Tween 20 PSCs. (**c**) J-V curves and (**d**) EQE spectra of champion pristine and 1.0 mM Tween 20 PSCs.

The schematic diagram and cross-section SEM image of the PSCs are shown in Figure 4a and Figure S3, respectively. It has the structure of an FTO/dense TiO_2_/mesoporous TiO_2_/MAPbI_3_ perovskite layer/spiro-OMeTAD/Ag electrode. The photovoltaic characteristics of the PSCs with various concentrations of Tween 20 in the perovskite films are shown in Figure 4b and Table 2. When the Tween 20 concentration increased from 0.0 to 1.0 mM, the short-circuit current density (J_SC_) increased from 19.89 to 21.46 mA/cm^2^, leading to a PCE of 15.75%. The improved photocurrent was due to the high absorption and uniform surface morphology (from Table S1) of the perovskite films with an appropriate amount of surfactant. On the contrary, the PCE of the PSCs with 3.0 mM Tween 20 remarkably decreased to 14.59%. When the Tween 20 concentration was increased to 3.0 mM, it led to the highest short-circuit current density (J_SC_) at 21.6 mA/cm^2^. The possible reason for the decrease in the PCE was the decreased crystal size due to an excess amount of Tween 20. The J-V curves of the champion pristine and 1.0 mM Tween 20 PSCs are shown in Figure 4c. For the pristine device, the J-V parameters were J_SC_ = 20.30 mA/cm^2^, V_OC_ = 1.031 V, FF = 69.3%, with a PCE of 14.51%, respectively. The maximum performances of the 1.0 mM Tween 20 PSC were J_SC_ = 22.90 mA/cm^2^, V_OC_ = 1.059 V, FF = 72.6%, with a PCE of 17.60%, respectively. The EQE spectra (Figure 4d) show that the integrated J_SC_ of the 1.0 mM Tween 20 PSC is 21.00 mA/cm^2^.

Herein, the thermal-assisted blade coating optimized the 1.0 mM Tween 20 perovskite films fabrication temperature to obtain the optimized grain size and device performance. It was found that the largest grain size and the best device efficiency were both obtained at 120 °C (Figure S4). This temperature was lower than the applied temperature of 130 °C for the one prepared without the Tween 20, as shown in our previous study [45,46]. This finding revealed that the addition of Tween 20 contributed to the growth of the perovskite crystal arrangement, leading to a lower optimal film-forming temperature.

**Figure 5:**
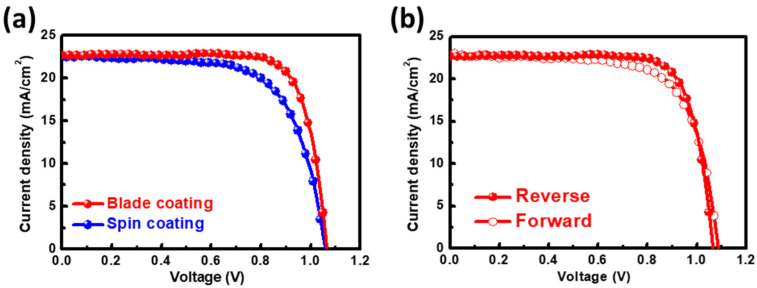
(**a**) J-V curves of the 1.0 mM Tween 20 PSCs prepared by the two different methods. (**b**) Hysteresis measurement of 1.0 mM Tween 20 PSCs prepared by the thermal-assisted blade-coating method.

To further illustrate the influence of the thermal-assisted blade-coating and spin-coating methods on the MAPbI_3_ perovskite device characteristics, the 1.0 mM Tween 20 perovskite films were prepared separately using the two different methods, and their surface morphologies are shown in Figure S5. Evidently, the thermal-assisted blade-coating method effectively reduced the grain boundaries of the perovskite films and had a larger domain size than that obtained by the spin-coating process. Figure 5a demonstrates the J-V curves of the perovskite device prepared by the two different methods. The spin-coated PSC exhibited a J_SC_ of 22.63 mA/cm^2^, V_OC_ of 1.060 V, FF of 66.92%, and PCE of 16.05%. In contrast, the blade-coated PSC achieved an efficiency of 18.80% and showed no apparent hysteresis (Figure 5b). 

**Figure 6:**
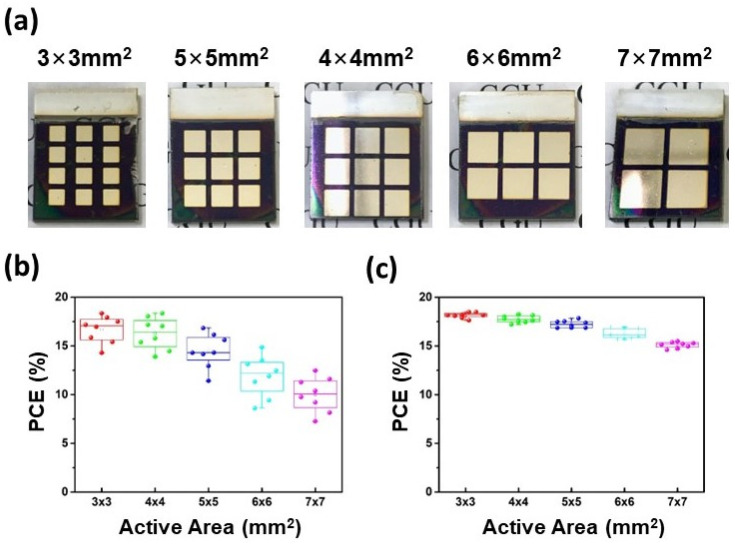
(**a**) Photos of perovskite devices with various active areas. (**b**) The area-dependent statistical performance of 1.0 mM Tween 20 PSCs fabricated by (**b**) the spin-coating method and (**c**) the thermal-assisted blade-coating method.

As previously mentioned, the thermal-assisted blade coating is suitable for fabricating large-area devices due to its coating uniformity. This advantage prompted us to fabricate PSCs with various active areas (Figure 6a), including 3 × 3, 4 × 4, 5 × 5, 6 × 6, and 7 × 7 mm^2^. The active areas versus the PCE of the 1.0 mM Tween 20 PSCs prepared by the two different methods are shown in Figure 6b,c. When the active area increased, the performance of the spin-coated PSC dropped dramatically, with a large standard deviation. However, the efficiency of large-area devices fabricated by the thermal-assisted blade-coating method was much more stable, meaning that this method produced high uniformity. These results were also in line with expectations.

**Figure 7:**
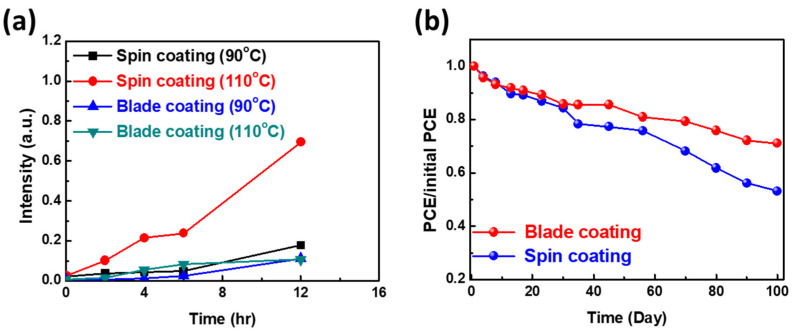
(**a**) Intensity ratio of PbI_2_ formed in the MAPbI_3_ perovskite film under thermal treatment and (**b**) long-term stability of PSCs fabricated by the two methods under ambient atmosphere (~45% relative humidity, 25 °C).

For the stability study, the 1.0 mM Tween 20 perovskite films prepared by the two methods were treated at 90 and 110 °C for 12 h, respectively. Both films were stable under 90 °C. However, the spin-coated film showed the significant production of a PbI_2_ peak, revealing that the perovskite film prepared by the thermal-assisted blade-coating method had less grain boundary and better thermal stability (Figure 7a). Moreover, the 1.0 mM Tween 20 PSCs prepared by the two different fabrication methods were exposed to the ambient atmosphere (∼45% relative humidity, 25 °C) for 100 days, as shown in Figure 7b. The PSC fabricated by the thermal-assisted blade-coating method retained 72% of its initial PCE after 100 days of exposure without encapsulation. In contrast, the spin-coated device only retained 53% of its initial PCE under the same condition. The excellent long-term stability reveals that perovskite film prepared by the thermal-assisted blade-coating method can reduce grain boundaries and further improve perovskite films’ crystal quality.

## 4. Conclusions

In conclusion, the effects of various concentrations of surfactant Tween 20 on MAPbI_3_ perovskite films prepared by thermal-assisted blade coating, including surface morphology, optical properties, crystal structure, and charge carrier dynamics, were systematically studied. The 1.0 mM Tween 20 perovskite film demonstrated a higher PL intensity and a longer carrier lifetime due to defect passivation. The champion PSC provided a PCE of 18.80%. Finally, the larger area PSC prepared by the thermal-assisted blade-coating method showed a higher performance and better ambient stability than the spin-coated devices.

## Figures and Tables

**Table 1:** Summary of the fast decay lifetime (τ_1_), the slow decay lifetime (τ_2_), and the average decay lifetime (τ_avg_) for MAPbI_3_ perovskite films/glasses.

Tween 20 Concentration (mM)	A (%)	τ_1_ (ns)	B (%)	τ_2_ (ns)	τ_avg_ (ns)
0.0	36.3	13.0	63.7	4.4	7.5
1.0	76.3	28.5	23.7	3.2	22.5
2.0	78.8	26.6	21.2	2.8	21.5
3.0	47.8	14.2	52.2	4.4	9.0

**Table 2:** Photovoltaic characteristics of pristine, 1.0, 2.0, and 3.0 mM Tween 20 PSCs. The average device parameters for 18 cells of same configuration are presented.

Tween 20 Concentration (mM)	J_SC_ (mA/cm^2^)	V_OC_ (V)	FF (%)	PCE (%)	Champion PCE (%)
0.0	19.89 ± 0.87	1.022 ± 0.020	67.8 ± 1.7	13.79 ± 0.82	14.51
1.0	21.46 ± 0.46	1.035 ± 0.022	70.9 ± 1.2	15.75 ± 0.62	17.60
2.0	21.02 ± 0.27	1.022 ± 0.025	69.5 ± 2.9	15.45 ± 0.75	16.23
3.0	20.03 ± 1.18	1.046 ± 0.023	69.6 ± 0.7	14.59 ± 0.96	14.94

## Data Availability

Not applicable.

## Supplementary Materials

The following supporting information can be downloaded at: https://www.mdpi.com/article/10.3390/nano12152651/s1. Figure S1: The XRD patterns of the perovskite films with different concentration Tween 20, and the reference standard for MAPbI3 and FTO; Figure S2: The grain size of perovskite films without and with different Tween 20 concentrations; Figure S3: FE-SEM images of cross-section PSC (a) without and with 1.0 mM Tween 20; Figure S4: The grain size of perovskite films with 1.0 mM Tween 20 with different fabrication temperatures; Figure S5: The top-view surface morphologies of perovskite film with 1.0 mM Tween 20 under two different preparation methods, including (a) spin coating and (b) thermal assisted blade coating method; Table S1: The morphological characteristics (Ra and RMS) of perovskite films with various Tween 20 concentrations by using AFM measurement.
