# Supplementary material for: Surfactant Tween 20 Controlled Perovskite Film Fabricated by Thermal Blade Coating for Efficient Perovskite Solar Cells
Source: Nanomaterials (Basel). 2022 Aug 2;12(15):2651. doi: 10.3390/nano12152651 (PMC9370445; doi:10.3390/nano12152651)
Supplement: Supplementary file 1 [file nanomaterials-12-02651-s001.zip › nanomaterials-1835733-supplementary.pdf]

Supplementary Information

Surfactant Tween 20 Controlled Perovskite Film Fabricated by Thermal Blade Coating for Efficient Perovskite Solar Cells

Kun-Mu Lee,[1,2,3,4*,‡] Shun-Hsiang Chan, [1,2*,‡] Chang-Chieh Ting,[5] Shih-Hsuan Chen,[1] Wei-Hao Chiu[4], Vembu Suryanarayanan[6], Jen-Fu Hsu[3], Ching-Yuan Liu[5*] and Ming-Chung Wu[1,2,3*]

1. Department of Chemical and Materials Engineering, Chang Gung University, Taoyuan 33302, Taiwan.
2. Green Technology Research Center, Chang Gung University, Taoyuan 33302, Taiwan.
3. Division of Neonatology, Department of Pediatrics, Chang Gung Memorial Hospital, Linkou, Taoyuan 33305, Taiwan.
4. Center for Reliability Sciences and Technologies, Chang Gung University, Taoyuan 33302, Taiwan.

5. Department of Chemical and Materials Engineering, National Central University, Jhongli District, Taoyuan 32031, Taiwan.

6. Electroorganic and Materials Electrochemistry Division, CSIR-Central Electrochemical Research Institute, Karaikudi 630003, India

‡K.-M. Lee and S.-H. Chan contributed equally to this work.

* Correspondence:

Kun-Mu Lee: e-mail, kmlee@mail.cgu.edu.tw
Ching-Yuan Liu: e-mail: cyliu0312@ncu.edu.tw
Ming-Chung Wu: e-mail, mingchungwu@cgu.edu.tw

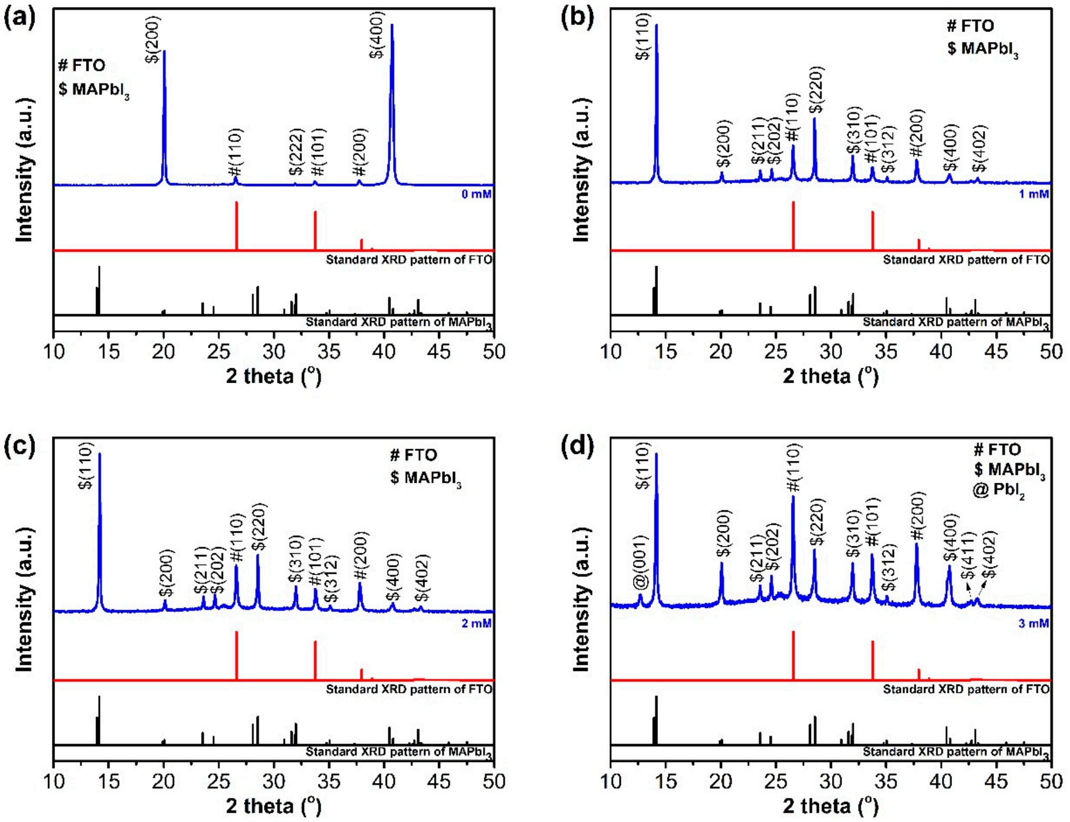

Fig. S1. The XRD patterns of the perovskite films with different concentration Tween 20, and the reference standard for $MAPbI_3$ and FTO.

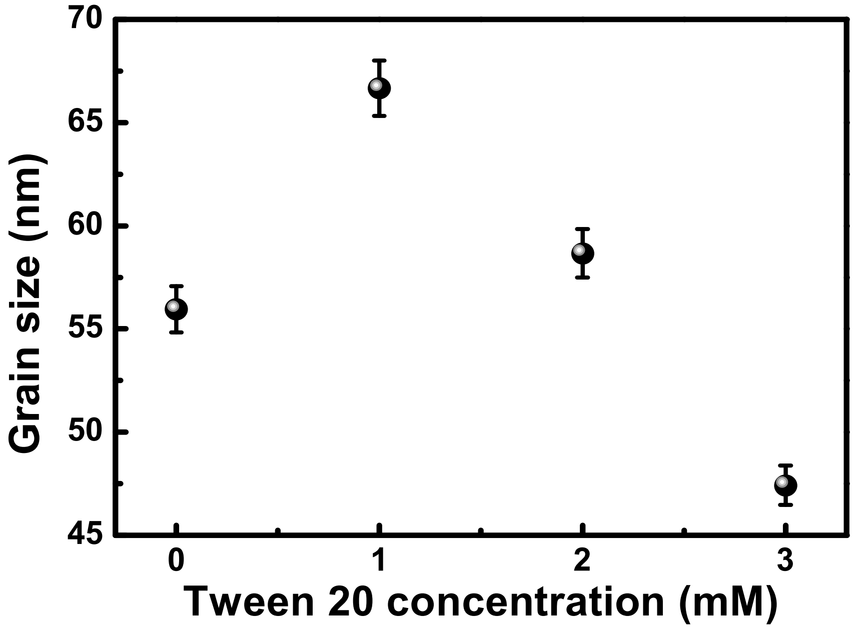

Fig. S2. The grain size of perovskite films without and with different Tween 20 concentrations.

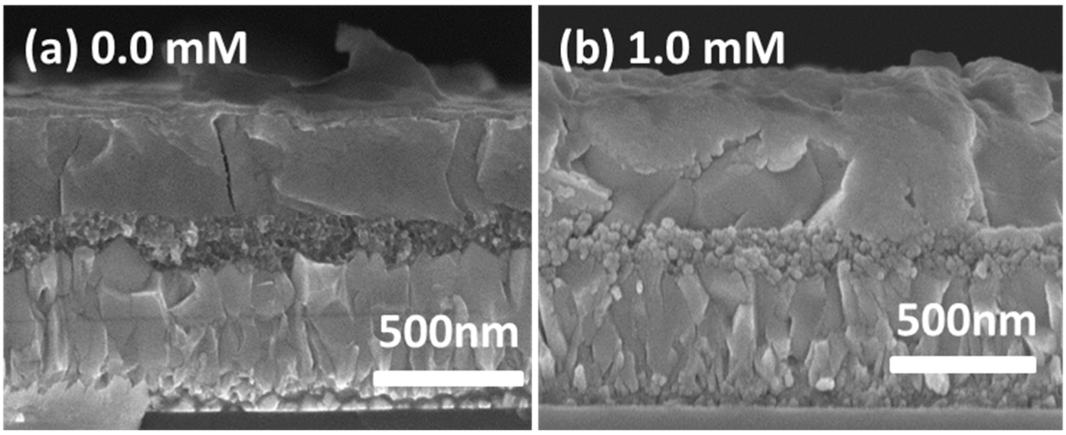

Fig. S3. FE-SEM images of cross-section PSC (a) without and with 1.0 mM Tween 20.

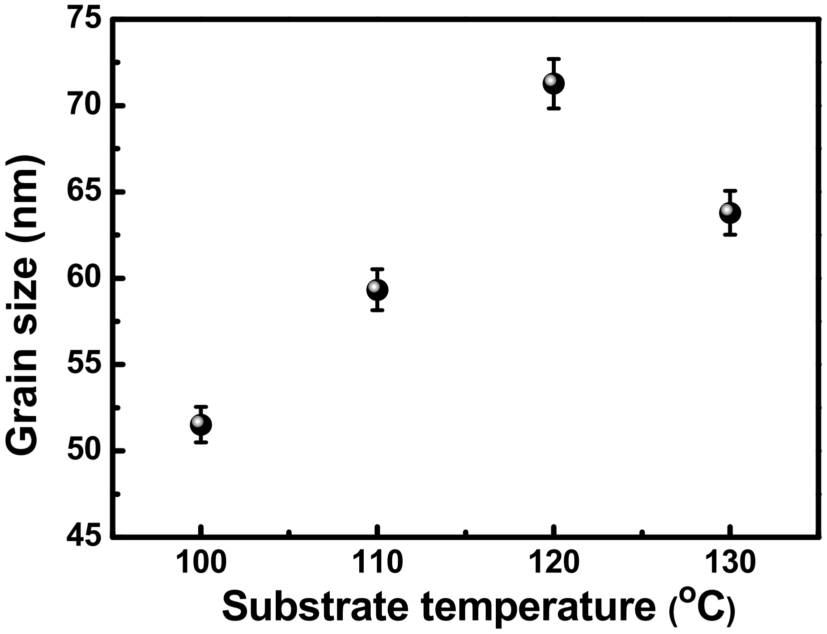

Fig. S4. The grain size of perovskite films with 1.0 mM Tween 20 with different fabrication temperatures.

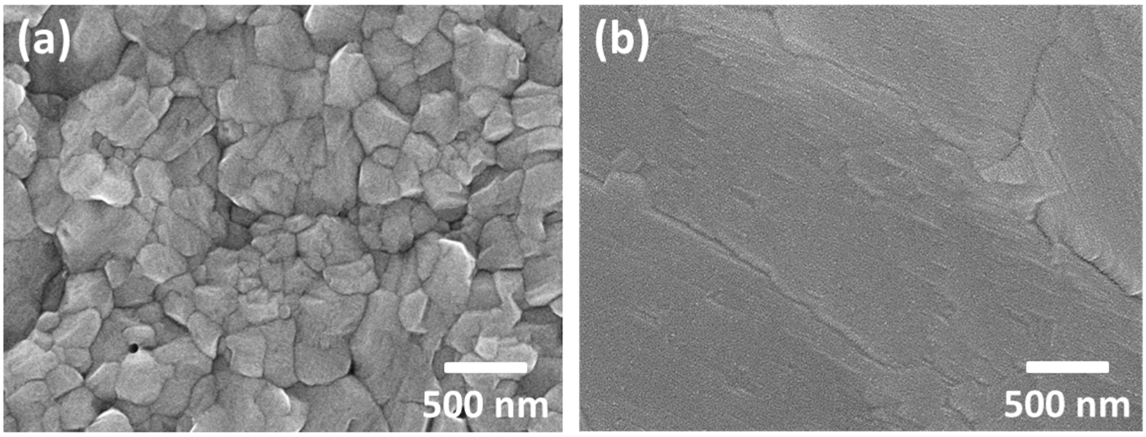

Fig. S5. The top-view surface morphologies of perovskite film with 1.0 mM Tween 20 under two different preparation methods, including (a) spin coating and (b) thermal assisted blade coating method.

Table. S1. The morphological characteristics (Ra and RMS) of perovskite films with various Tween 20 concentrations by using AFM measurement.

Concentration of Tween 20 (mM)	Ra (nm)	RMS (nm)
0	110.3	140.5
1	52.1	67.9
2	103.6	130.6
3	120.7	148.8
